# Topoisomerase II– and Condensin-Dependent Breakage of *MEC1^ATR^*-Sensitive Fragile Sites Occurs Independently of Spindle Tension, Anaphase, or Cytokinesis

**DOI:** 10.1371/journal.pgen.1002978

**Published:** 2012-10-25

**Authors:** Nadia Hashash, Anthony L. Johnson, Rita S. Cha

**Affiliations:** Division of Stem Cell Biology and Developmental Genetics, National Institute for Medical Research, Medical Research Council, London, United Kingdom; National Cancer Institute, United States of America

## Abstract

Fragile sites are loci of recurrent chromosome breakage in the genome. They are found in organisms ranging from bacteria to humans and are implicated in genome instability, evolution, and cancer. In budding yeast, inactivation of Mec1, a homolog of mammalian ATR, leads to chromosome breakage at fragile sites referred to as replication slow zones (*RSZ*s). *RSZ*s are proposed to be homologous to mammalian common fragile sites (CFSs) whose stability is regulated by ATR. Perturbation during S phase, leading to elevated levels of stalled replication forks, is necessary but not sufficient for chromosome breakage at *RSZ*s or CFSs. To address the nature of additional event(s) required for the break formation, we examined involvement of the currently known or implicated mechanisms of endogenous chromosome breakage, including errors in replication fork restart, premature mitotic chromosome condensation, spindle tension, anaphase, and cytokinesis. Results revealed that chromosome breakage at *RSZ*s is independent of the *RAD52* epistasis group genes and of *TOP3*, *SGS1*, *SRS2*, *MMS4*, or *MUS81*, indicating that homologous recombination and other recombination-related processes associated with replication fork restart are unlikely to be involved. We also found spindle force, anaphase, or cytokinesis to be dispensable. *RSZ* breakage, however, required genes encoding condensin subunits (*YCG1*, *YSC4*) and topoisomerase II (*TOP2*). We propose that chromosome break formation at *RSZ*s following Mec1 inactivation, a model for mammalian fragile site breakage, is mediated by internal chromosomal stress generated during mitotic chromosome condensation.

## Author Summary

Chromosome breakage can occur during normal cell division. When it occurs, the breaks do not arise randomly throughout the genome, but at preferred locations referred to as fragile sites. Chromosome breakage at fragile sites is an evolutionarily conserved phenomenon, implicated in evolution and speciation. In humans, fragile site instability is also implicated in mental retardation and cancer. Despite its biological and clinical relevance, the mechanism(s) by which breaks are introduced at mammalian fragile sites remains unresolved. Although several plausible models have been proposed, it has not been possible to ascertain their contribution, largely due to the lack of a suitable experimental system. Here, we study a yeast model system that closely recapitulates the phenomenon of chromosome breakage at mammalian fragile sites. We eliminate all but one of the currently considered models—premature compaction of the incompletely replicated genome in preparation for their segregation during cell division. We also find that the breakage required functions of three proteins involved in the genome compaction, an essential process that is evolutionarily conserved from bacteria to humans. Our findings suggest that a fundamental chromosomal process required for normal cell division can paradoxically cause genome instability and/or cell death, by triggering chromosome breakage at fragile sites.

## Introduction

Unintended double strand breaks (DSBs) arise during the unchallenged life of the cell. These breaks do not arise randomly throughout the genome, but occur preferentially at loci referred to as fragile sites. Fragile sites exist in all organisms examined to date including bacteria, yeast, flies, plants, and mammals. Examples include the bacterial *ter* [1], budding yeast replication slow zones (*RSZ*s) [2], and mammalian common- and rare- fragile sites [3], [4]. Some fragile sites are loci of specialized DNA/chromosomal processes. For example, the bacterial *ter* function as preferred loci of replication fork termination [5]. For the majority of fragile sites however, their precise function, assuming that it exists, remains elusive.

The term “fragile site” was first used to describe a heritable locus of recurrent chromosome breakage on metaphase spreads of human lymphocytes [3]. Currently, there are more than 120 fragile sites identified in the human genome [6]. Notably, not all fragile sites form breaks at the same frequency, and some are more prone to breakage than others. For example, *FRA3B* at 3p14.2 is the most fragile site in the human genome, exhibiting breaks in 50% of metaphases after a mild replication stress [6], [7]. Reason(s) for the differential tendencies for breakage among mammalian fragile sites is not known.

Mammalian fragile sites are classified as either rare or common, depending on their frequency within the population. Rare fragile sites are seen in <5% of the population. Most rare fragile sites are tri-nucleotide repeats, whose increased breakage is caused by expansion of the repeats [6]. Common fragile sites (CFSs), on the other hand, are present in every chromosome and in all individuals. Furthermore, common fragile sites are conserved throughout mammalian evolution [8], [9] suggesting that they might be a normal component of the chromosome [4].

Mammalian fragile sites are said to be “expressed” when they display signs of breaks or gaps on metaphase chromosome spreads. Studies have identified several conditions that may play a role in mammalian fragile site expression. These include: (i) the time at which a locus is replicated during normal S phase, based on the early observations that the vast majority of mammalian fragile sites replicate late [10]; (ii) mild inhibition of DNA replication, contributing to elevated levels of stalled replication forks and further delays in the replication of the normally late replicating fragile loci [11]; (iii) inactivation of checkpoint proteins such as ATR [12] or ATM [13]; (iv) inactivation of proteins involved in DSB repair and/or replication fork restart [14]; (v) premature onset of mitosis [15]; and (vi) anaphase and/or cytokinesis [16], [17]. These observations led to a number of models regarding the mechanism underlying fragile site expression. In all cases, replication fork stalling is proposed to be the initiating event, with stalled forks ultimately giving rise to a DSB by a process or processes whose exact nature remains unresolved. The uncertainly is, in part, due to the fact that equally plausible hypotheses have not yet been tested in a suitable model system.

The budding yeast genome, like that of other organisms, contains different types of fragile sites. These differ with respect to their structure, distribution in the genome, and genetic requirement for their stability or breakage [2], [18]–[23]. The Replication Slow Zone (*RSZ*) is a fragile site that was identified based on its sensitivity to the loss of Mec1 function [2]. Mec1, like its mammalian counterpart ATR, is an essential protein [24] involved in a number of fundamental processes, including genome duplication, DNA repair, recombination, meiosis, and checkpoint regulation [24]–[27]. It promotes dNTP synthesis during every G1-S transition to ensure that the cell has sufficient levels of dNTPs for genome duplication [28], [29]. Mec1 up-regulation of dNTP synthesis is also essential during replication stress- or DNA damage- checkpoint responses [30], [31]. Additional checkpoint functions of Mec1 include stabilization of stalled forks, coordination of repair, and preventing cell cycle progression until the damage situation is resolved [27], [32], [33].

The name *RSZ* was based on the observation that replication forks moved notably slower through these regions than through other loci during normal S phase [2]. In *MEC1* cells, forks continue to progress through *RSZ*s, eventually completing their duplication. In *mec1-4* cells, replication forks progress more or less normally until they reach *RSZ*s. At *RSZ*s, the forks remain stalled for about 90 minutes, until the appearance of DSBs at these loci some time during G2/M [2]. Analysis of eleven *RSZ*s identified on chromosomes III and VI suggests that they do not occur randomly along the chromosome, but occur between highly active replication origins along the entire length of the chromosome; a notable exception, however, is the centromeric region, which lacks a *RSZ* [2; N Hashash and R Cha, unpublished data].

*RSZ*s and mammalian CFSs are both large genetic determinants, each comprising about 0.1% of the respective genome (i.e. ∼10 kb *RSZ* of 1.5×10^7^ bp budding yeast genome and ∼1 Mb CFS of the 3×10^9^ bp mammalian genome). Some studies reported a correlation between the occurrence of some CFSs or *RSZ*s and certain features of the genome, including high flexibility, high AT content, hairpin structure, and/or hotspots for ectopic genome integration [2], [6], [34], [35]. Currently however, there are no structural or functional features that can be utilized for their *a priori* identification. *RSZ*s and CFSs are both late replicating loci [2], [10] and exhibit sensitivity to mild replication stress or deficiencies in Mec1 or ATR [2], [12], [19]. Largely based on these similarities, *RSZ*s were proposed to be homologous to mammalian CFSs [2], [19], [35].

Here, we investigate the mechanism of *RSZ* breakage following Mec1 inactivation. Specifically, we tested involvement of each of the five processes implicated in mammalian fragile site expression (above). Results showed that *RSZ* breakage following Mec1 inactivation required functions of topoisomerase II (Top2) and the condensin complex; in the absence of Top2 or condensins, *RSZ*s did not break, even though replication forks still stalled. In contrast, replication fork restart, spindle tension, anaphase, or cytokinesis were all dispensable for *RSZ* breakage. Based on these observations, we propose that internal chromosomal stress, generated during mitotic chromosome condensation, promotes the conversion of stalled forks at *RSZ*s to DSBs.

## Results

### Key genes required for processing of stalled replication forks are dispensable for *RSZ* breakage

Replication forks stall during unchallenged S phase either as a part of normal replication program [2], [21], [36], [37] or incidentally, upon encountering a damaged template [33] or due to insufficient levels of dNTPs [19], [30], [32]. In either case, the resumption of DNA synthesis from stalled forks or the rescue by the firing of cryptic origins, while maintaining the integrity of stalled forks, is essential for cell's survival. Homologous recombination plays a key role in replication fork restart [38]. During this process, DSBs can be generated as an intermediate and contribute to endogenous chromosome breakage. To test whether breakage at *RSZ*s is generated via homologous recombination or recombination-related process, we utilized a previously characterized temperature sensitive *mec1* strain, *mec1-4* [2], and assessed the effects of eliminating relevant proteins; homologous recombination proteins (Rad50, Rad51, Rad52, Rad54, Rad55, and Mre11), the Sgs1^BLM^-Top3 complex, the Srs2 helicase, and the Mus81-Mms4 endonuclease, a putative resolvase [38]–[42]. Thermal inactivation of Mec1-4 results in prolonged replication fork stalling at *RSZ*s, followed by chromosome breakage at these loci. The breaks appear typically around 90–120 minutes after alpha-factor arrest/release, corresponding to the G2/M phase of the first cell cycle after the release.

**Figure 1:**
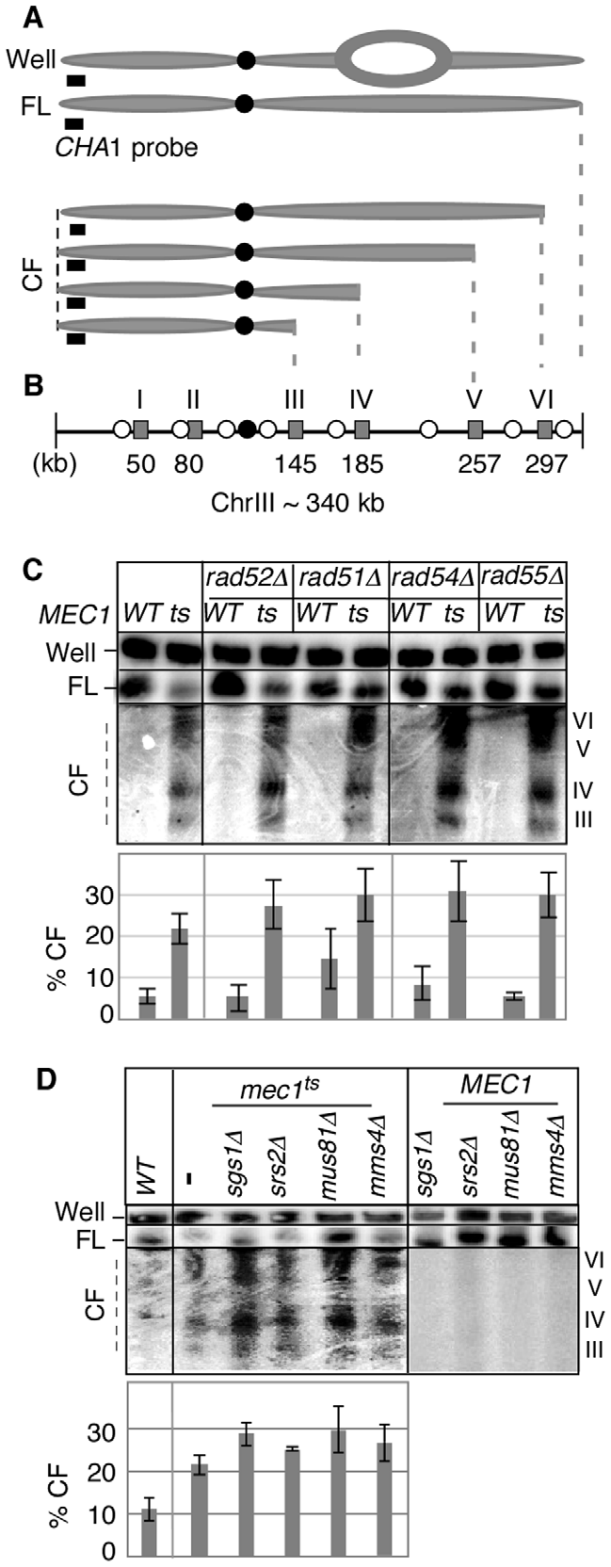
Key components of fork-restart process are dispensable for *RSZ* breakage. (A) ChrIII species revealed by PFGE (pulse field gel electophoresis) followed by labelling of one chromosome end using *CHA1* as a probe. The approach allows for detection of full-length linear chromosome (“FL”), nonlinear forms (e.g. replication bubbles) that remain in the wells of the gel (“Well”), and linear chromosome fragments extending from the labelled end (“CF”) [2]. (B) Distribution of six *RSZ*s, referred to as *RSZ*-I through *RSZ*-VI, on ChrIII [2]. Depicted are: *RSZ*s (grey rectangle), active origins (open circle), and centromere (filled circle). The approximate mid-point of each *RSZ* in kb is as indicated. In a typical PFGE/Southern analysis using the *CHA1* probe detects *RSZ*s III through VI. (C, D) Strains deleted for the indicated genes in either *MEC1* (“WT”) or *mec1-4* (“ts”) were released from alpha-factor arrest at 23°C into YPD at 37°C, a restrictive temperature for *mec1-4*. Samples were collected 3 hours after the release and assessed for *RSZ* breakage by PFGE/Southern methods described (Materials and Methods). *CHA1* hybridization signal from the Well, FL, and CF regions were quantified using Image J software. Depicted in the graph is the fraction of signal associated with CF in each lane. In (C), the graph shows the average level of breakage from at least three independent experiments; error bars denote +/− one standard error of the mean. In (D), the graph shows the average level of breakage from two independent experiments; error bars denote the lower and higher values observed for each strain.

*MEC1* and *mec1-4* strains carrying a null allele of one of the genes mentioned above were arrested with a-factor at 23°C and released into fresh YPD media at 37°C, a restrictive temperature for *mec1-4* [2]. Samples were collected 3 hours after the release, and the status of chromosome III (ChrIII) was assessed by pulse field gel electrophoresis (PFGE) followed by Southern hybridization using a telomere-proximal probe, *CHA1* (Figure 1A, 1B). As shown previously [2], DSBs enriched for *RSZ*s in ChrIII were observed in the *mec1-4* culture (Figure 1C, 1D). Elimination of various proteins with a role in the replication fork restart did not prevent break formation at *RSZ*s (Figure 1; data not shown) indicating that the involvement of this process was unlikely.

### Inappropriate mitosis does not contribute to *RSZ* breakage

The spindle assembly checkpoint (SAC) is an evolutionarily conserved mechanism responsible for ensuring that every pair of sister-chromatids is under spindle tension prior to anaphase. The SAC monitors this process by assessing microtubule occupancy of the kinetochores and/or tension generated across chromosomes/kinetochores [43]. Like the other checkpoint systems, the SAC is a signal transduction cascade and is mediated by the Mad1, 2, 3 (mitotic arrest deficient) and Bub1, 2, 3 (budding uninhibitied by benzimidazole) proteins. In the absence of these proteins, cells proceed through mitosis irrespective of whether all sister kinetochores are under spindle tension, resulting in frequent chromosome mis-segregation and cell death.

Although the SAC was originally thought to operate independently of the DNA damage checkpoint, recent evidence suggests an interplay between the two. For example, Mec1/Tel1 have been shown to inhibit anaphase by utilizing Mad/Bub proteins independently of the kinetochores in response to DNA damage [44], which might account for the earlier observation that about 50% of *mec1-4* cells undergo mitosis despite the presence of unresolved replication forks [2]. These considerations raise the possibility that *RSZ* breakage might occur as a result of inappropriate mitosis. If this was the case, we reasoned that inactivation of the SAC might increase *RSZ* breakage by allowing a greater proportion of *mec1-4* cells to proceed through mitosis with unresolved replication forks at *RSZ*s. We tested this possibility by assessing the impact of deleting *MAD2* or *BUB2* on *RSZ* breakage.

**Figure 2:**
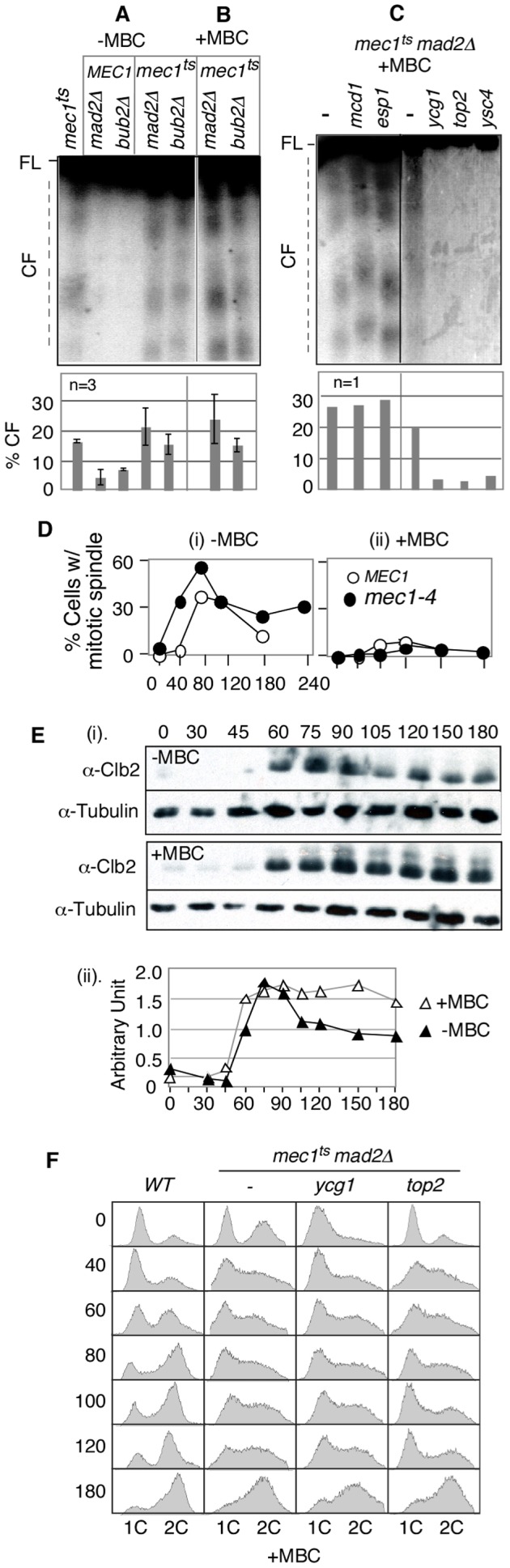
*RSZ* breakage is independent of spindle tension but requires Top2 and condensin components. (A–C) Strains of indicated genotypes were released from alpha-factor arrest at 23°C into YPD in the absence (A) or presence of a spindle poison MBC (B and C) at 37°C (a restrictive temperature for all conditional alleles utilized). Samples were collected 3 hours after the release and assessed for *RSZ* breakage by PFGE/Southern methods (Figure 1A; Materials and Methods). In (A) and (B), the graph shows the average level of breakage from at least three independent experiments; error bars denote +/− one standard error of the mean. In (C), the graph shows the level of chromosome breakage observed in the image presented. (D) *MEC1* and *mec1-4* strains were released from alpha-factor arrest into YPD at 37°C in the presence (ii) or absence (i) of MBC. Samples were collected at the indicated time points and assessed for the status of spindles using an anti-tubulin antibody. Shown in the graph is the fraction of cells containing spindles greater than 2 µm. At least 100 cells were analyzed in each sample. (E) A *mec1-4* culture was released from alpha-factor arrest into YPD at 37°C in the presence or absence of MBC. Samples were collected at the indicated time points and assessed for Clb2 levels. Signals from Clb2 and the tubulin bands were quantified using ImageJ software, and the fraction of Clb2 signal at each point is shown on the graph. (F) Strains of indicated genotypes were released from alpha-factor arrest into YPD+MBC media at 37°C. Samples were collected at various time points and subjected to fluorescence activated cell scan (FACS) analysis (Materials and Methods).

*MEC1* and *mec1-4* strains in *mad2Δ BUB2*, *MAD2 bub2Δ*, or *MAD2 BUB2* backgrounds were arrested with alpha-factor at 23°C and released into fresh YPD media at a restrictive temperature for *mec1-4*. Samples were collected 3 hours after the release, and the status of ChrIII was assessed. As expected, *RSZ* breakage was observed in *mec1-4* control culture (Figure 2A). *RSZ* breakage was also observed in the *mec1-4 mad2Δ* or *mec1-4 bub2Δ* cultures (Figure 2A). Furthermore, the extent of breakage was comparable in the presence or absence of *MAD2/BUB2*, suggesting that *RSZ* breakage was unlikely to be caused by compromised SAC function, leading to inappropriate mitosis.

### Spindle tension is dispensable for *RSZ* breakage

Another implication of the lack of an impact of *mad2Δ* or *bub2Δ* is that *RSZ* breakage might be independent of spindle tension. Assuming that inactivation of the SAC would have allowed some cells to proceed through mitosis in the absence, or with a reduced level, of spindle tension, we reasoned that *mad2Δ* or *bub2Δ* may have resulted in a reduction in *RSZ* breakage if spindle tension played a role. To directly address the involvement of spindle tension, we investigated the effects of microtubule depolymerising drugs such as methyl1-2-benzimidazolecarbamate (MBC) or nocodazole. First, we confirmed that spindle poison was effective in preventing elongation of spindles in *mec1-4* cells (Figure 2D). In the absence of spindle poison, the elongation in *mec1-4* cells occurred reproducibly earlier than in *MEC1* (Figure 2Di). The reason for this remains unknown but is likely to be related to the role(s) of Mec1/Tel1/Rad53 in regulating spindle status in response to DNA damage or replication stress [45], [46]. We also found that MBC blocked Clb2 degradation, a readout for mitotic exit [47], in *mec1-4* (Figure 2E). The latter suggested that although *mec1-4* cells were compromised in preventing the onset of mitosis in the presence of stalled forks [2], they were competent in mediating a spindle damage-dependent SAC response.

To ensure that we assessed the impact of spindle depolymerisation on *RSZ* breakage, rather than the impact of SAC response to the depolymerisation, we decided to examine the effects of spindle poison in the absence of the SAC. *mec1-4 mad2*Δ or *mec1-4 bub2*Δ strains were released from alpha-factor arrest as described above, except that they were released into YPD media containing either MBC or nocodazole (Materials and Methods). Samples were collected 3 hours after the release and assessed for *RSZ* breakage. Results showed chromosome breakage in the presence of either drug (Figure 2B; data not shown), demonstrating that mitotic spindles are dispensable for *RSZ* breakage.

### *RSZ* breakage requires Top2 and condensin function

The dispensability of mitotic spindles prompted us to consider whether internal chromosomal stress might be involved in *RSZ* breakage. During mitotic prophase, the duplicated genome undergoes dramatic structural reorganization, leading to sister chromatid individualisation and chromosome compaction in preparation for segregation during anaphase [48], [49]. To test whether the intra-chromosomal stress generated during these processes might have a role in *RSZ* breakage in the absence of spindle tension, we assessed the impact of inactivating relevant gene products. These included; (i) Scc1/Mcd1 (hereon referred to as Scc1), a component of the cohesion complex that holds sister chromatids together until their disjunction at the onset of anaphase [47], [50], (ii) Esp1, a caspase-like cysteine protease that promotes sister chromatid separation by mediating the cleavage of Scc1 [51], (iii) Ycg1 and Ysc4, two non SMC components of the condensin complex, required for mitotic chromosome compaction [52], [53], and (iv) Top2, a type II topoisomerase that catalyzes decatetation of DNA strands between the sister chromatids to allow their resolution and facilitate chromosome condensation [48], [49], [54].

A set of *mec1-4 mad2*Δ strains, each expressing temperature sensitive *scc1*, *esp1*, *ycg1*, *ysc4*, or *top2* alleles were released from alpha-factor arrest into YPD + MBC media at 37°C, a restrictive temperature for all of the conditional alleles utilized. Samples were collected 3 hours after the release and analysed for *RSZ* breakage (Figure 2C). As expected, *RSZ* breakage was observed in the *mec1-4 mad2*Δ control strain. The breakage was also observed in strains expressing a temperature sensitive *scc1* or *esp1* allele, suggesting that *RSZ* breakage occurred independently of the status of the cohesins. In contrast, inactivation of Top2, Ycg1, or Ysc4 suppressed chromosome breakage, suggesting that mitotic chromosome condensation might be involved in *RSZ* breakage in the absence of spindle tension.

To rule out the possibility that the *top2*, *ycg1*, or *ysc4* suppression was mediated by their impact on S phase progression, either by allowing replication forks to progress through *RSZ*s [2] or by committing cells to inviability before forks reach a *RSZ* [19], we assessed their impact on the status of S phase progression. A WT, *mec1-4 mad2*Δ, or *mec1-4 mad2*Δ strain expressing a temperature sensitive allele of either *top2* or *ycg1* was released from G1 arrest into YPD+MBC media at 37°C. Samples were collected at various time points after the release and subjected to fluorescent activated cell scan (FACS) analysis (Materials and Methods). In a WT control, cells proceeded through S phase and completed bulk genome duplication by 80 minutes following alpha-factor arrest/release (Figure 2F). In contrast, S phase progression in a *mec1-4 mad2*Δ culture, like that in a *mec1-4* culture [2] was delayed (Figure 2F), consistent with earlier observations that the status of *MAD2* did not confer any effects on DNA replication [55]. Thermal inactivation of *top2* or *ycg1* did not exert a notable effect on S phase progression, suggesting that the *top2*, *ycg1*, or *ysc4* suppression was unlikely to be due to their impact on DNA replication. Taken together, we conclude that Top2/condensin-mediated mitotic chromosome condensation triggers *RSZ* breakage in the absence of mitotic spindles.

### *RSZ* breakage in the presence of spindle tension also requires Top2 and condensin function

**Figure 3:**
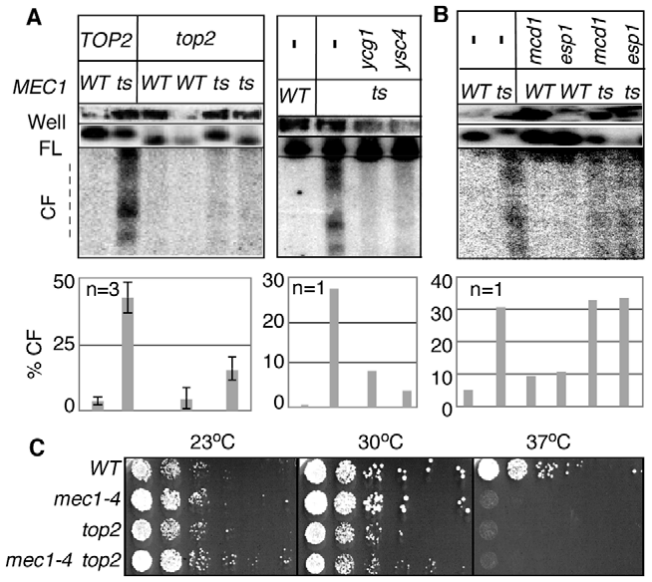
*RSZ* breakage in the presence of spindle tension requires Top2 and condensin components. (A, B) Strains of indicated genotypes were released from alpha-factor arrest into YPD without any spindle poison at a restrictive temperature. Samples were collected 3 hours after the release and analysed for *RSZ* breakage (Materials and Methods; Figure 1A). For *top2* analysis, results of two different *top2 MEC1* and *top2 mec1-4* strains are presented. The graph shows the average level of breakage from at least three independent experiments. Error bars denote +/− one standard error of the mean. For *ygc1*, *ysc4*, *scc1*, and *esp1*, the graph shows the average level of breakage from two independent experiments. Error bars denote the lower and higher values observed for each genotype. For *ygc1*, *ysc4*, and *scc1*, two different strains of the same genotype were analyzed; for *esp1*, the same strain was analyzed in two independent experiments. (C) The indicated strains were grown to log phase at 23°C in YPD. Each culture was diluted to an OD_600_ of 0.4 and 10-fold serial dilutions were spotted onto YPD agar. The agar plates were then incubated at the indicated temperatures for three days.

Results thus far showed that breakage of *RSZ*, like that of mammalian CFSs, can occur in the absence of spindle tension. Importantly however, breakage of *RSZ*s or CFSs occurs during normal cell proliferation in the presence of spindle tension. Thus, it was formally possible that the Top2/condensin dependent *RSZ* breakage was a mechanism operating specifically in the absence of mitotic spindles. To address this, we assessed the effects of inactivating Top2, Ycg1, or Ysc4 in the presence of spindle tension. A set of *mec1-4 MAD2 BUB2* strains expressing temperature sensitive alleles of *top2*, *ycg1*, or *ysc4* was released from G1 arrest into a fresh YPD media in the absence of spindle poison. Samples were collected 3 hours after the release and analysed for *RSZ* breakage. The results showed that *top2*, *ycg1*, or *ysc4* suppressed *RSZ* breakage (Figure 3A). In contrast, thermal inactivation of Scc1 or Esp1 did not prevent the breakage (Figure 3B). These results suggest that the genetic requirement (and the mechanism, by extension) of *RSZ* breakage in the presence or absence of spindle tension is likely to be the same. We conclude that Top2/condensin mediated mitotic chromosome condensation is required for *RSZ* breakage during normal cell proliferation irrespective of the status of spindle tension.

As expected from the essential nature of *TOP2* and condensin, inactivation of these gene products did not rescue the lethality of *mec1-4* at non-permissive temperature (Figure 3C; data not shown).

### *RSZ* breakage occurs independently of anaphase or cytokinesis

**Figure 4:**
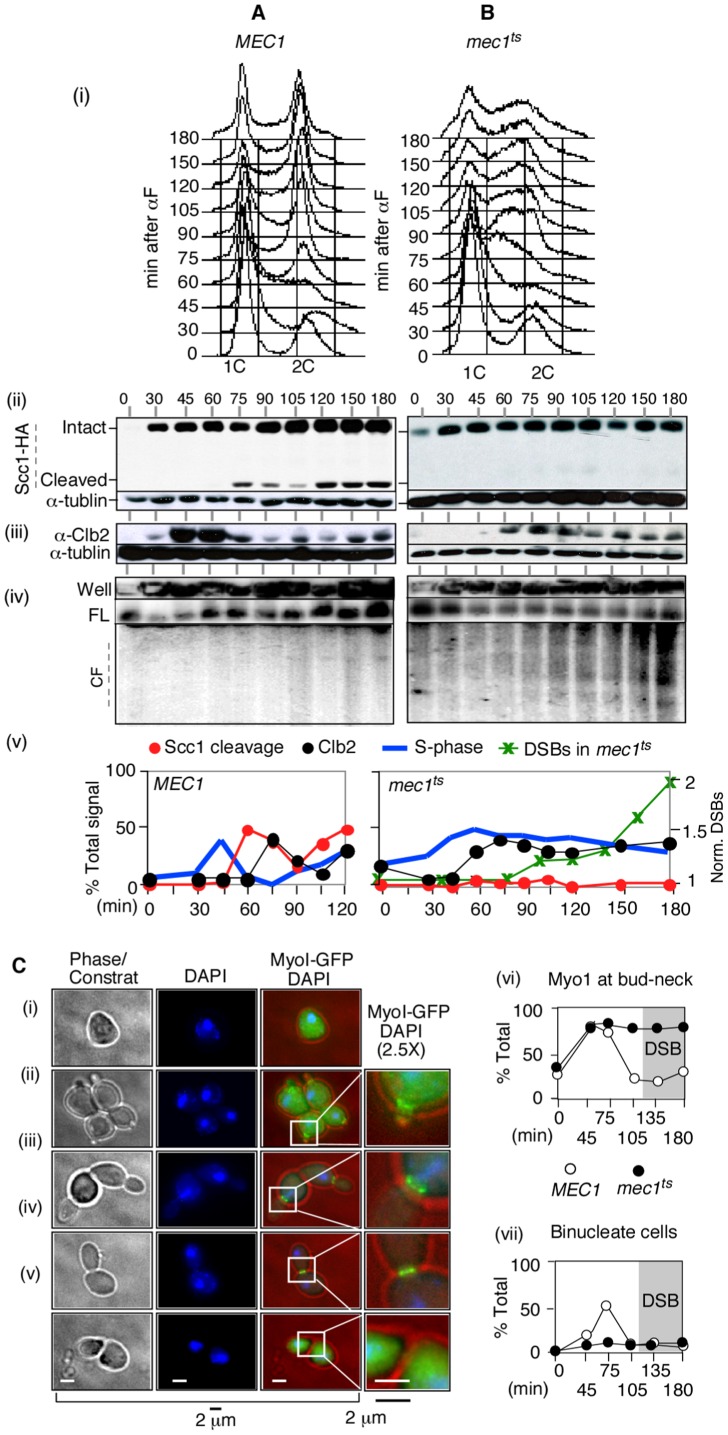
*RSZ* breakage in the presence of spindle tension occurs independent of anaphase, mitotic exit, or cytokinesis. (A, B) Samples from alpha-factor released cultures of *MEC1 SCC1-HA* (A) and *mec1-4 SCC1-HA* (B) strains were collected at the indicated time points and analyzed for S phase progression by FACS analysis (i), cohesin cleavage (ii), Clb2 levels (iii), and chromosome breakage (iv). (v) Signals associated with Scc1-HA, Cleaved Scc1-HA, and Clb2 at each time point were quantified and normalized to those of tubulin. The “% Signal” for Scc1-cleavage (open circle) is defined as the amount of cleaved Scc1-HA signal divided by the sum of Scc1-HA and cleaved-Scc1-HA at each time point, whereas that for Clb2 (closed circle) is defined as the amount of Clb2-signal divided by that of tublin. The proportion of cells in S phase was quantified as described [73]. DSBs: *CHA1* signals from Well, FL, and CF regions in the PFGE/Southern analysis (iv) were quantified and the fraction of CF-signal was calculated relative to the total signal for each time point. These values were normalized to the maximum value observed at t = 180 minutes. (C) Alpha-factor arrested cultures of *MEC1 MYO1-GFP* and *mec1-4 MYO1-GFP* strains were released into YPD at 30°C, a restrictive temperature for *mec1-4*. To limit the cells to the first cell cycle, alpha-factor was added back to the culture 45 minutes after release. Samples were collected at the indicated time points for fluorescence microscopy. (i–v) Cellular morphology at various stages in cell cycle. Samples taken from a *MEC1 MYO1-GFP* culture undergoing synchronous cell division cycle was examined for bud morphology, nuclear division (“DAPI”), and onset of cytokinesis (loss of “Myo1-GFP” at the bud neck [“*”]). Representative images of cells at different stages: (i) G0/G1, (ii) S phase, (iii) G2, (iv) following genome segregation but before cytokinesis, and (v) post cytokinesis. (vi,vii) Fraction of cells with Myo1-GFP signal (comprising of categories ii, iii, and iv) and those that have undergone genome segregation (“binucleate”, iv) in *MEC1* and *mec1-4* cells as a function of time. In the *mec1-4* culture, DSBs began to accumulate starting at t = 120 minutes (data not shown).

If *RSZ* breakage is independent of spindle tension, then a prediction might be that it should also be independent of the events downstream of the SAC execution point, such as anaphase, mitotic exit, and cytokinesis. We tested this by monitoring the occurrence of these events in *mec1-4* and *MEC1* cultures as they proceeded through a synchronous cell cycle in the presence of spindle tension. Samples were collected at various time points following alpha-factor arrest/release and assayed for *RSZ-*expression, Scc1-cleavage, a readout for the onset of anaphase [43], and Clb2-degradation, a readout for exit from mitosis [47] (Figure 4).

In the *MEC1* culture, cells completed bulk genome duplication between 60–75 minutes following alpha-factor release (Figure 4A panels i and v). An Scc1 cleavage product was observed starting from 75 minutes after release (Figure 4A panels ii and v). Levels of Clb2 peaked at 45 and 60 minutes following the release and decreased rapidly thereafter (Figure 4A, panels iii and v). These results indicate that, in the *MEC1* culture, the completion of bulk genome duplication (60–75′), onset of anaphase (75–90′), and exit from mitosis (90′) occurred in a temporally ordered manner. PFGE/Southern analysis of ChrIII showed that chromosome breakage in this culture remained at background levels (Figure 4A panel iv).

In the *mec1-4* culture, the cells remained stuck in mid-S phase from about 60 minutes following the release, suggesting that anaphase did not take place (Figure 3B panels i and v). Scc1 cleavage was not observed (Figure 3B panels ii and v). A modest reduction in Clb2 levels was observed starting 90 min after the release, although the extent of reduction was notably less than that observed in the *MEC1* culture (Figure 3B, panels iii and v). In this *mec1-4* culture, DSBs began to accumulate starting at t = 90–105 minutes (Figure 3B panels iv and v). These results demonstrate that *RSZ*-expression in the presence of spindle tension occurs in the absence of Scc1 cleavage, Clb2 degradation, or, by extension, the onset of anaphase or exit from mitosis, respectively.

Next, the occurrence of cytokinesis was assessed. To this end, we generated *MEC1* and *mec1-4* strains expressing an endogenous copy of *MYO1-GFP*, and monitored the appearance of binucleate cells with or without the Myo1-GFP signal (Figure 4C panel iv versus panel v). *MYO1* encodes a component of the actomyosin ring that localizes to the bud neck from early S phase (e.g. Figure 4C panel ii). The Myo1 ring remains at the neck until cytokinesis, during which the ring constricts and Myo1 disappears from the bud neck [56] (Figure 4C panel v). *MEC1 MYO1-GFP* and *mec1-4 MYO1-GFP* cells were released from alpha-factor arrest into fresh YPD media at a restrictive temperature in the absence of spindle poison. Samples were collected at various time points and assessed for the morphology of DNA (via a DAPI stain) and the status of Myo1-GFP ring. In both cultures, the Myo1-GFP ring appeared by 45 minutes after release (Figure 4C panel vi). In *MEC1* cells, the Myo1-GFP ring remained at the bud neck until 75 minutes and disappeared by 105 minutes, indicating that cytokinesis had occurred. In contrast, the Myo1-GFP ring in *mec1-4* cells remained at the bud neck throughout the duration of the experiment, indicating that cytokinesis did not take place (Figure 4C panel vi). In this culture, the fraction of cells that had undergone anaphase – i.e. those containing two DAPI staining bodies that were separated by MyoI-GFP ring (e.g. Figure 4C panel iv) remained low, in agreement with the lack of Scc1 cleavage (Figure 4Bii). In the *MEC1* control, the fraction of binucleate cells reached about 50% by t = 75 minutes; thereafter, the fraction decreased as the cells underwent cytokinesis. *RSZ* expression in the *mec1-4* culture was observed at t = 120 minutes (data not shown).

Taken together, these results show that *RSZ* breakage in the presence of spindle tension occurs independently of anaphase, mitotic exit, or cytokinesis. The simplest interpretation would be that the breakage occurs before the onset of anaphase.

## Discussion

The aim of this study was to examine the mechanism of chromosome breakage at *RSZ*, a *MEC1-*sensitive fragile site and a model for mammalian CFSs. Specifically, we tested involvement of the following possibilities, each of which had been implicated in mammalian fragile site expression: (i) errors in replication fork restart,; (ii) premature mitotic chromosome condensation; (iii) spindle tension; (iv) anaphase; or (v) cytokinesis. Evidence revealed that Top2 and condensin proteins were required for *RSZ* breakage; in contrast, the key proteins involved in replication fork restart, spindle tension, anaphase, or cytokinesis were dispensable.

In all eukaryotes examined to date, an essential function of Top2 and condensins is chromosome compaction [48], [49], [53], [54], [57], [58]. Although the extent of mitotic chromosome condensation in budding yeast is about two orders of magnitude less than that observed in metazoan cells (the compaction ratio is 160 in yeast versus 10,000–20,000 in metazoans; 57,58), inactivation of budding yeast Top2 or condensin subunits also results in a chromosome compaction defect [52], [59], [60], in agreement with the notion that the mechanism is evolutionarily conserved.

**Figure 5:**
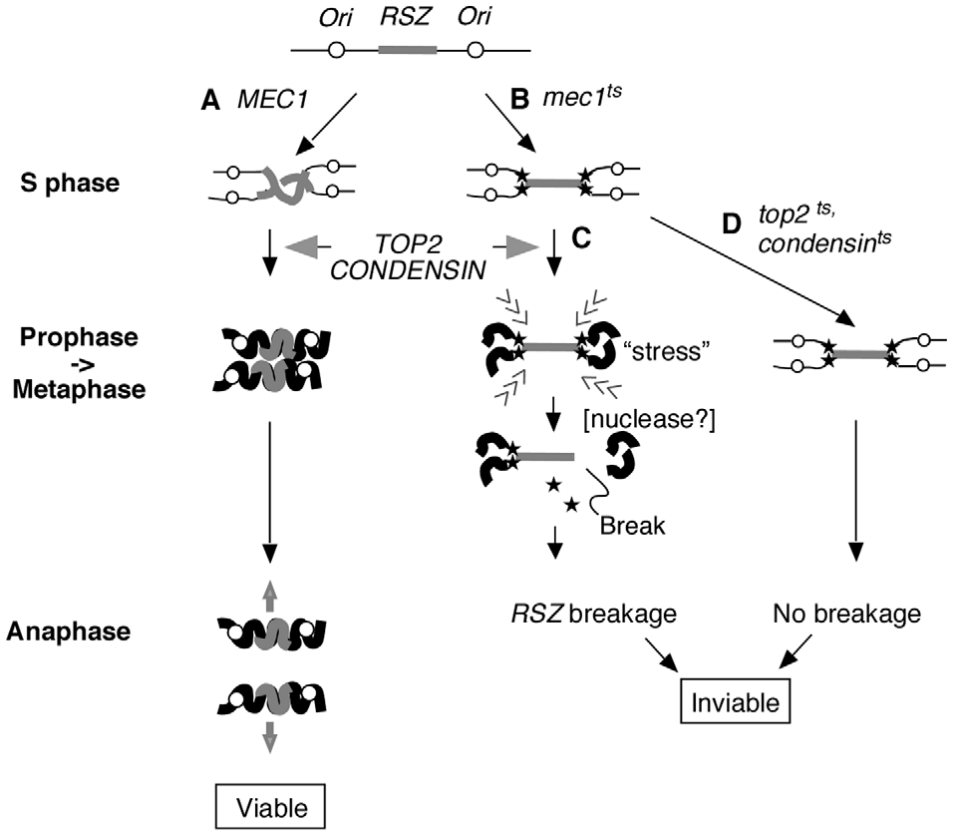
Proposed mechanism of *RSZ* breakage. *RSZ*s are loci of delayed replication during normal S phase found in alternation with active replication origins (Figure 1B) [2]. (A) In a *WT* strain, the duplicated genome undergoes Top2- and condensin-mediated chromosome condensation during mitotic prophase in preparation for its segregation at anaphase. (B) In the absence of Mec1 function, replication forks (filled stars) stall at *RSZ*s. Despite the stalled forks, the cells exit S phase and proceed through mitotic prophase. (C) In the presence of Top2 and condensin, the incompletely duplicated genome is subjected to chromosome compaction during which intra-chromosomal stress triggers *RSZ* breakage. The breakage is independent of spindle tension and cohesin status, and occurs before the onset of anaphase. The molecular mechanism responsible for converting stalled forks to DSBs remains unknown but is likely to involve a nuclease (see text). (D) In a *top2* or condensin mutant, the partially duplicated genome is not subjected to mitotic condensation and the breakage does not occur. Nevertheless the cells lose viability likely due to the lack of an essential Top2 or condensin function(s).

Taken together, we propose a model whereby two temporally and genetically distinguishable events mediate chromosome breakage at *RSZ*s (Figure 5). In WT cells, the genome duplication is complete by the end of S phase (Figure 5Ai). During mitotic prophase, the duplicated genome undergoes Top2- and condensin- dependent chromosome compaction (Figure 5Aii) in preparation for its disjunction during anaphase (Figure 5Aiii). In the absence of Mec1 function, replication forks stall at *RSZ*s (Figure 5Bi); despite this, the cells exit S phase and proceed through the cell cycle. During prophase, the incompletely duplicated genome of *mec1-4* cells becomes subjected to Top2- and condensin- dependent chromosome compaction (Figure 5Bii). We propose that internal stress generated during this process promotes the conversion of stalled forks to a DSB. The molecular mechanism underlying the catalysis of breakage is unknown, but may involve a nuclease that is yet to be identified (below). Above evidence indicates that chromosome breakage is independent of spindle tension or tension-dependent events such as anaphase or cytokinesis. This breakage is also independent of the status of sister chromatid cohesins, consistent with the report that cohesion removal, while essential for sister chromatid resolution, is dispensable for mitotic chromosome compaction [61]. In the absence of Top2 or condensins (Figure 5C), chromosome condensation does not take place; therefore, the incompletely duplicated genome of *mec1-4* cells is not subjected to the internal stress that triggers the conversion of stalled forks to DSBs. Nevertheless, the cells die, likely due to the lack of an essential Top2 or condensin function(s) [52], [62].

With regard to the dispensability of the replication fork restart process, it is important to note that the list of candidate genes examined is not exhaustive, and therefore, we cannot rigorously eliminate its involvement based on this line of evidence. Nevertheless, our results unequivocally rule out the involvement of some of the key players in replication fork restart that had previously been implicated in breakage at different types of fragile sites (see below); the *RAD52* epistasis group proteins, the Sgs1^BLM^-Top3 complex, the Srs2 helicase, and the Mus81-Mms4 endonuclease [38]–[42].

The dispensability of spindle tension is not surprising in the light of the fact that the distribution pattern of *RSZ*s is different from that of the spindle tension mediated breaks. Specifically, *RSZ*s are found between active replication origins along the entire length of the chromosome except for the centromeric region ([2]; N. Hashash and R. Cha, unpublished data). In contrast, spindle tension-dependent DSBs tend to occur around the centromere, the locus of greatest spindle tension [22], [63]. Mammalian CFSs, like *RSZ*s, are found along the chromosome arms. Furthermore, the fact that mammalian fragile sites are defined as loci of recurrent breaks or gaps on metaphase chromosome spreads, obtained from cultures treated with spindle poisons such as colchicines [3], [4], strongly suggest that expression of mammalian fragile sites, like that of *RSZ*s, occurs independently of spindle tension.

The amount of force exerted by a pair of microtubules at the centromere (i.e. 20 piconewton [pN]) is estimated to be at least an order of magnitude smaller than that required to break the chromosome (i.e. 480 pN) [64], [65]. Assuming that the intra-chromosomal stress generated during mitotic chromosome compaction is less than that generated by the spindles, it is likely that the Top2/condensin-dependent *RSZ* breakage is mediated by an endonuclease. As a means to test whether Top2 was the responsible enzyme, we performed Top2 ChIP-on-CHIP analysis in *MEC1* and *mec1-4* cells, reasoning that if Top2 catalyzed the cleavage, we might observe its enrichment at *RSZ*s. Analysis thus far has failed to show any such enrichment, suggesting that its direct involvement was unlikely (N Hashash, R Cha, Y Katou, K Shirahege; unpublished data). Nevertheless, this observation alone does not eliminate the possibility, because Top2 may dissociate from the ends of the DSB after DNA cleavage, and therefore would not normally remain enriched at *RSZ*s. Alternatively, the cleavage might be mediated by a different protein, for example, Yen1, an evolutionarily conserved Holiday junction resolvase that is activated during M phase of the cell division cycle [66] or proteins involved in post replication repair [67]. It is also possible that the DSBs at *RSZ*s result from cleavage of single stranded DNA associated with stalled forks [68].

A positive role for Top2 and condensin in chromosome breakage is unexpected in light of the observations that their inactivation caused, rather than prevented, DSB formation [e.g. [22], [23], [69], [70]. Also surprising is the dispensability of anaphase or cytokinesis in *RSZ* breakage. Upon a closer examination, however, it becomes apparent that the chromosome breakage examined in each study was at different types of fragile loci in the genome, in that they differed with respect to their structure (e.g. a hairpin or a specific protein-DNA complex), distribution (e.g. chromosome arms versus the centromeres) and/or the timing of their expression (e.g. during S phase, before anaphase, or during cytokinesis) [2], [16], [18]–[20], [22], [23], [41], [69], [71]. These observations provide further support for the notion that both the stability and the expression of each type of fragile sites is under a specific genetic and regulatory control [19].

Among the different types of fragile sites identified and characterized to date, the *RSZ* appears to be the closest structural and functional homolog of mammalian fragile sites. Furthermore, among the currently proposed mechanisms of mammalian fragile site expression, the mechanism of *RSZ* breakage inferred in the current study seems to be most consistent with the original definition of a mammalian fragile site, that it is a heritable locus of recurrent breaks or gaps on metaphase chromosome spreads [3]. Taken together, it is tempting to speculate that the mechanism of *RSZ* breakage and that of CFS expression, at least for those that are sensitive to the loss of ATR or ATM functions [12], [13], might be conserved and that the mammalian Top2 and condensin may similarly play a role in promoting fragile site expression.

## Materials and Methods

### Yeast strains and media

All strains were of the SK1 background except those noted. Relevant genotypes of the strains are listed in Table S1. Unless specified otherwise, cells were grown in YPD (1% [w/v] yeast extract, 2% [w/v] bacto-peptone, 2% [w/v] glucose) media. To obtain a synchronous culture for cell cycle analysis, mid-log cultures were arrested with 5 µg/ml alpha-factor for 3 hours before being released to fresh YPD media. For temperature-sensitive strains, cells were arrested with alpha-factor at 23°C before releasing to YPD media prewarmed to a restrictive temperature. To induce microtubule deploymerization, cells were grown in the presence of either 15 µg/ml nocodazole (Sigma-Aldrich) or 40 µg/ml carbendazim (MBC; Sigma-Aldrich).

### Fluorescence-activated cell scan (FACS)

Cells from 1 ml of relevant samples were fixed (40% [v/v] ethanol, 0.1 M sorbitol) for at least 3 hours before being pelleted, resuspended in RNase solution (50 mM Tris-HCl pH 7.5, 100 µg/ml RNaseA) and incubated overnight at 37°C. The next day, the cells were treated with 500 µl of pepsin solution (50 mM HCl, 5 mg/ml pepsin) for a minimum of 5 minutes at room temperature, before being resuspended in 1 ml SYTOX solution (50 mM Tris-HCl pH 7.5, 1 µM SYTOX Green; Invitrogen Molecular Probes). The samples were incubated overnight at 4°C. The next day, they were analyzed on a Becton Dickinson FACSan using Cell Quest software (Becton Dickinson).

### Chromosome breakage analysis by pulse field gel electrophoresis (PFGE)/Southern blot analysis

Chromosome-sized DNA in agarose plugs for PFGE was prepared as described [72]. Electrophoresis was performed at 14°C in a Bio-Rad CHEF Mapper under the following condition: a voltage gradient of 6 V/cm, switch times of 5–30 sec, a switch angle of 115°, in a 1% agarose gel in 0.5× TBE for 24 hours. The DNA in gels was transferred to nylon membranes and hybridized with ^32^P-labeled *CHA1* probe, a 757 bp HindIII-BamHI fragment (−156 to +601 of the ORF) restricted from a pUC19 based plasmid (pRSC38). The image was visualized and signals quantified using a Storm 860 PhosphorImager and ImageJ software, respectively.

### Fluorescence microscopy

900 µl from appropriate samples was incubated with 100 µl 37% (w/v) formaldehyde (Fisher Scientific) for 10 minutes at room temperature. The cells were pelleted and washed twice with 1 ml PBS, and then resuspended in 200 µl PBS. A 10 µl sample of the cell suspension was spread onto a glass microscope slide and left to dry. Before application of the glass coverslip, 2 µl of 4,6-diamino-2-phenylindole (DAPI; Sigma) solution (1.5 µg/ml in Vectashield mounting medium [Vector Lab]) was dotted onto the dried cells. Fluorescence microscopy was performed on a Deltavision Spectris system.

### Western blots

Whole-cell extracts were prepared from cell suspension in 20% trichloroacetic acid by agitation with glass beads. Precipitated proteins were solubilized in SDS-PAGE sample buffer and appropriate dilutions were subjected to SDS-PAGE and Western blotting. Antibodies utilized for Western blotting were, rabbit polyclonal anti-Clb2 (Santa Cruz Biotechnology Inc), mouse monoclonal anti-HA (12CA5; NIMR, London), mouse monoclonal anti-MYC (9E10; NIMR, London), and rat monoclonal anti-tubulin (YL1/2; Abcam). For each antibody a 1∶1000 dilution was used for Western Blotting except for anti-tubulin, which was used at a 1∶5000 dilution.

## Supporting Information

Table S1 Strains utilized in current study.
(DOC)
