# Supplementary material for: Topoisomerase II– and Condensin-Dependent Breakage of MEC1ATR-Sensitive Fragile Sites Occurs Independently of Spindle Tension, Anaphase, or Cytokinesis
Source: PLoS Genet. 2012 Oct 25;8(10):e1002978. doi: 10.1371/journal.pgen.1002978 (PMC3486896; doi:10.1371/journal.pgen.1002978)
Supplement: Table S1 — Strains utilized in current study. (DOC) [file pgen.1002978.s001.doc]

**Table S1. Strains utilized in current study**

Relevant genotype Strains^1^  Source^2^

| *WT* | 234, RCY307 |  |
| --- | --- | --- |
| *rad50∆* | RCY1187, RCY1204 |  |
| *rad51∆* | 254 |  |
| *rad52∆* | 249 |  |
| *rad54∆* | 247 |  |
| *rad55∆* | RCY693 |  |
| *mre11∆* | RCY2527 |  |
| *sgs1∆* | 113 |  |
| *srs2∆* | 226 | Neil Hunter |
| *mus81∆* | RCY2414 | Neil Hunter |
| *mms4∆* | RCY2416 | Neil Hunter |
| *bub2∆* | 450 |  |
| *mad2∆* | 451 |  |
| *scc1-73* | FK385 | Frank Uhlmann |
| *esp1-1* | 389 | Doug Koshland |
| *top2-1* | 30, 31, 440 | John Nittis |
| *top3∆* | RCY2412 |  |
| *ycg1-2* | 430 | Doug Koshland |
| *ycs4-2* | 432 | Doug Koshland |
| *mec1-4* | 204, 362 |  |
| *mec1-4, rad50∆* | RCY1185 |  |
| *mec1-4, rad51∆* | 256 |  |
| *mec1-4, rad52∆* | 251 |  |
| *mec1-4, rad54∆* | 257,  RCY708 |  |
| *mec1-4, rad55∆* | RCY737 |  |
| *mec1-4, mre11∆* | RCY1488 |  |
| *mec1-4, sgs1∆* | 206 |  |
| *mec1-4, srs2∆* | 207 |  |
| *mec1-4, mus81∆* | RCY728 |  |
| *mec1-4, mms4∆* | RCY732 |  |
| *mec1-4, bub2∆* | 448 |  |
| *mec1-4, mad2∆* | 410 |  |
| *mec1-4, scc1-73* | RCY2839, 2841 |  |
| *mec1-4, esp1-1* | 385 |  |
| *mec1-4, top2-1* | 34, 35, 41, 442 |  |
| *mec1-4 top3∆* | RCY760 |  |
| *mec1-4, ycg1-2* | 380, 381 |  |
| *mec1-4, ycs4-2* | 383, 384 |  |
| *MYO1-GFP* | 449, 471, 472 |  |
| *mec1-4, MYO1-GFP* | 470, 473, 474, 466 |  |
| *SCC1-3xHA* | RCY2025 |  |
| *mec1-4, SCC1-3xHA* | 296 |  |
| *mec1-4, mad2∆,  scc1-73* | RCY2871 |  |
| *mec1-4, mad2∆,  esp1-1* | 477 |  |
| *mec1-4, mad2∆,  top2-1* | RCY2807, 2808 |  |
| *mec1-4, mad2∆,  ycg1-2* | RCY2832, 2833 |  |

^1^. NHY is the prefix for all strains utilized unless specified otherwise.

^2^. If gift from other laboratories.
